# Development and validation of a PEST-based instrument for assessing macro-environmental factors in Olympic athletes' preparation under crisis conditions

**DOI:** 10.3389/fspor.2026.1856666

**Published:** 2026-06-25

**Authors:** Olga Kuvaldina, Geiziane Leite Rodrigues Melo, Asta Sarkauskiene, Larysa Taran

**Affiliations:** 1 Health Research and Innovation Science Centre, Klaipeda University, Klaipeda, Lithuania; 2 Department of Theoretical Foundation of Olympic and Professional Sports, Admiral Makarov National University of Shipbuilding, Mykolaiv, Ukraine; 3 Department of Olympic and Professional Sports, Kharkiv State Academy of Physical Culture, Kharkiv, Ukraine

**Keywords:** content validity, Olympic sport, PEST analysis, questionnaire validation, Ukraine

## Abstract

**Introduction:**

Understanding how external crises influence elite sport requires context-specific research tools. This study aimed to develop and validate two structured questionnaires based on the PEST framework (Political, Economic, Socio-cultural, and Technological) to evaluate macro-environmental factors affecting the preparation of Ukraine's Olympic team in times of instability.

**Materials and methods:**

A panel of seven experts in sport science from four countries assessed 55 items (27 for Tokyo 2020 and 28 for Paris 2024) across three dimensions: clarity, relevance, and appropriateness. Ratings were collected using a 5-point Likert scale. The Content Validity Coefficient (CVC) was used to measure item-level agreement. Items scoring below 0.80 were revised and re-assessed after cognitive interviews.

**Results:**

Post-revision, all items achieved acceptable CVC values (≥0.80). The average CVC ranged from 0.80 to 1.00, with minimal variation between raters. Expert feedback improved item clarity and relevance. The instrument demonstrated strong content validity.

**Discussion:**

The content-validated tools provide a way to assess how political and systemic disruptions affect Olympic preparation. They are ready for further application in longitudinal studies focused on the national sport system in crisis contexts.

## 1 Introduction

In recent decades, the landscape of high-performance sport has become increasingly complex. Scholars typically distinguish between macro-level structures—such as policy, funding, and governance—and micro-level environments like coaching, training load, and athlete development (1–4). These levels interact within a dynamic global system that has passed through several phases: initial internationalization, escalating financial competition, and more recently, a growing focus on innovation, technology, and social impact (5, 6).

Major global disruptions—particularly the COVID-19 pandemic—have revealed how elite sport is closely linked to political and economic systems. During such crises, sport has functioned as both a strategic policy tool and a symbol of national resilience (7). Rapid organizational adaptation, digital innovation, and new value-generation models emerged in response to unstable conditions (8). These experiences underscore the vulnerability of national sport systems to external shocks.

According to authors (9), National Olympic Committees (NOCs) face crises triggered by either internal mismanagement or powerful external forces. The latter—such as pandemics, budget cuts, or armed conflicts—require urgent institutional response despite originating beyond the organization's control. These “outside forces crises” threaten not only operational continuity but also reputational capital and athlete support systems.

In Ukraine, these external shocks converged dramatically. The COVID-19 pandemic and the full-scale war with Russia have together disrupted facilities, dislocated athletes, and strained institutional resources (10–14). Given these overlapping crises, relying solely on traditional performance indicators is insufficient. Decision-makers require structured tools to understand how macro-level pressures impact Olympic preparation and long-term athlete development.

Recent work on Olympic governance emphasizes the need for sport organizations to anticipate and respond to crisis conditions (9). The PEST framework—covering political, economic, socio-cultural, and technological factors—offers a useful structure for examining such external pressures in elite sport. Within sport management, political factors relate to governance, sport policy, and crisis-related institutional decisions; economic factors include funding, resource allocation, and financial constraints; socio-cultural factors cover athlete wellbeing, social support, displacement, and public attitudes; and technological factors concern access to training technologies, digital tools, and innovation in preparation processes (1, 3, 9, 15). Together, these dimensions provide a systematic basis for examining macro-environmental pressures on Olympic preparation under crisis conditions.

While interest of macro-environmental analysis in sport has grown (16–19), few tools exist to assess these pressures systematically. This gap is especially pressing in crisis contexts where decisions must be grounded in evidence. Existing research rarely offers validated instruments that can measure the external conditions affecting Olympic preparation.

Although macro-level factors such as politics, economics, and technology are increasingly acknowledged in elite sport research, validated tools to assess them remain limited (15, 20–22). In the Ukrainian context, no validated instrument exists to track how crises—like pandemics or war—disrupt Olympic preparation. A structured tool based on the PEST framework is therefore needed to capture these external influences systematically.

This study applies the PEST analytical framework to examine how external disruptions affect Olympic sport systems. As a first step, content validity was established using the Content Validity Coefficient (CVC) (23, 24), which is well-suited for small expert panels and adjusts for chance agreement (25).

Two PEST-based questionnaires were developed and validated to assess external pressures on Ukraine's Olympic preparation for Tokyo 2020 and Paris 2024. The instruments were tested through a stepwise process: expert scoring, cognitive interviews, and review by an international panel. This study produced content-validated instruments for identifying crisis-related disruptions and informing policy responses in elite sport.

## 2 Materials and methods

### 2.1 Instrument development

The instruments under validation are two structured questionnaires based on the PEST framework (Political, Economic, Socio-cultural, and Technological factors), designed to assess external influences on the preparation of the Ukrainian National Olympic Team under crisis conditions. The first questionnaire includes 27 items related to the Tokyo 2020 Olympic cycle, while the second includes 28 items related to the Paris 2024 cycle (Supplementary Appendis A and B). Separate questionnaires were developed because the two cycles reflected different crisis contexts: the Tokyo cycle was primarily shaped by pandemic-related disruptions, whereas the Paris cycle occurred under conditions of prolonged war and martial law. Although both instruments shared the same PEST structure, separate versions were needed to capture cycle-specific external pressures.

Item development followed a stepwise process. First, literature on elite sport systems and policy factors (1, 3, 15), Olympic governance under crisis conditions (9), and macro-environmental/PEST analysis in sport (17–19) was reviewed to identify external factors relevant to Olympic preparation under crisis conditions. These factors were organized according to the PEST framework and translated into questionnaire items reflecting political, economic, socio-cultural, and technological influences. The initial item pool was discussed during a preliminary expert panel involving the authors and Ukrainian specialists in Olympic sport and sport science. This discussion helped refine item wording, clarify domain allocation, and ensure contextual relevance before the formal content validation procedure.

Each item in both questionnaires was evaluated along three dimensions: clarity of wording, appropriateness to the PEST domain, and relevance to the construct being measured. Each dimension was rated on a 5-point Likert scale (1—not at all, 5—completely) (26).

**Figure 1:**
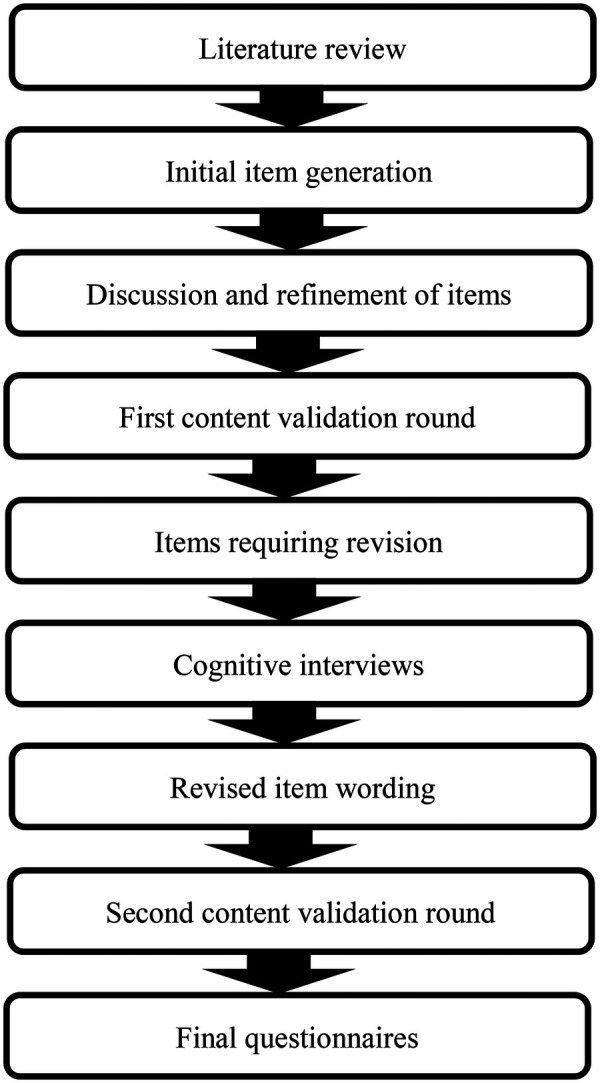
Development and content validation process of the PEST-based instruments.

The overall development and content validation process of the PEST-based instruments is summarized in Figure 1.

To assess the content validity of the questionnaires, seven experts were purposefully selected for their professional qualifications and extensive experience in sport science, elite athlete preparation, and Olympic sport. Before the evaluation, each expert completed a brief profile documenting their academic background, institutional affiliation, and years of experience. All experts hold doctoral degrees in sport science and academic titles, with professional experience ranging from 20 to over 40 years. The international composition of the panel (Germany, Lithuania, Poland, and Ukraine) ensured strong contextual and methodological diversity.

Ethical procedures were strictly followed. Experts were contacted individually by email and provided with detailed information about the study's purpose and procedures; participation was voluntary, and informed consent was implied by questionnaire completion. To minimize bias, the evaluation was conducted blindly, and experts were unaware of other panel members. Each received a personalized link and completed the assessment independently.

A reminder email was sent after two weeks, and optional consultations were offered to clarify procedural questions without influencing the ratings. No personal identifiers were collected, and all data were processed anonymously.

A summary of the expert panel is provided in Table 1.

**Table 1:** Profile of experts participating in the content validation panel.

Expert	Country	Affiliation	Role/Area of Expertise	Years of Experience
1	Germany	German Sport University Cologne	PhD, Senior lecturer Institute of Sport History/Olympic Studies Centre	25
2	Lithuania	Klaipeda University	PhD, Professor, sport science	20
3	Poland	Branch of AWF in Biala Podlaska	PhD, Professor, sport science	20
4	Ukraine	National Olympic Committee of Ukraine	PhD, Professor, sport science	28
5	Ukraine	Kharkiv State Academy of Physical Culture	Habilitated doctor, Professor, sport science	25
6	Ukraine	Admiral Makarov National University of Shipbuilding	PhD, associated professor, sport science	38
7	Ukraine	Admiral Makarov National University of Shipbuilding	PhD, associated professor, sport science, Honored coach of Ukraine	25

### 2.2 Procedure

Each expert independently evaluated all items in both questionnaires according to three criteria—clarity, appropriateness, and relevance—using a standardized electronic form. Open-ended comments were also invited to support qualitative refinement.

Content validity was quantified using the Content Validity Coefficient (CVC) proposed by Hernández-Nieto (23). For each item and criterion, the CVC was calculated as:

$$CVC_{i}=\frac{{\bar{X}}_{i}-1}{K-1}-V_{ies}$$

where: ${\bar{X}}_{i\;}$, is the mean score given by the experts; K = 5 is the maximum score on the scale.

$V_{ies}$ reflects the degree of disagreement among experts. Lower values indicate higher consensus, while higher values may suggest ambiguity or divergence in interpretation.

$$V_{ies}={\left(\frac{1}{m}\right)}^{m}$$

*m =* 7 is the number of experts.

$$V_{ies}=1.2\times 10^{-6}$$

For the entire instrument:

$$CVC_{t}=\frac{1}{n}\overset{n}{\underset{i=1}{\sum }}CVC_{i}$$

*n—*is the number of items in the questionnaire.

This continuous metric (0–1) reflects the degree of consensus, with values ≥0.80 indicating acceptable content validity (25). To complement the CVC analysis, standard deviations and coefficients of variation were computed for each item across the three criteria as indicators of inter-rater variability or potential bias. All calculations were performed in Microsoft Excel. Items falling below the CVC threshold were flagged for revision.

### 2.3 Targeted cognitive interviewing

To refine items with sub-threshold content validity (CVC < 0.80), a targeted cognitive interviewing procedure was conducted. This method, widely recognized in survey research, is effective for detecting problems in item comprehension and interpretation (27, 28). A sub-sample of experts from the initial validation panel participated in individual online interviews focused only on items that did not reach the CVC threshold. Experts were asked to explain how they understood each item, identify unclear wording, and suggest alternative formulations. The authors compared the experts' comments and revised items when similar concerns were repeated or when the suggested wording improved clarity and alignment with the relevant PEST domain. The revised items were then submitted to the second validation round.

### 2.4 Second round of expert validation

Following the cognitive interviews and item revisions, a second content validation round was undertaken. The same panel of seven experts independently reassessed the modified items using the established criteria of clarity, appropriateness, and relevance on the original 5-point scale (23, 25). Updated CVC values were calculated for each dimension, complemented by standard deviation and coefficient of variation to evaluate the consistency of expert judgements. This iterative procedure enabled empirical confirmation of improvements in item clarity and construct alignment.

### 2.5 Ethical approval

The study received a positive evaluation from the Klaipeda University Biomedical Research Scientific-Ethical Committee under the Health Research and Innovation Science Centre. The Committee confirmed that the study meets all applicable ethical requirements. Expert participation was voluntary and anonymous, and no personal or sensitive data were collected. The research complies with institutional and national ethical standards.

## 3 Results

### 3.1 Content validity assessment for questionnaire items evaluating PEST factors affecting the preparation of the Ukrainian Olympic team for the games of the XXXII olympiad in Tokyo

Table 2 summarizes the expert-based content validity results for the first questionnaire assessing external (PEST) factors influencing the preparation of the Ukrainian National Olympic Team for the Games of the XXXII Olympiad (Tokyo 2020). All 27 items were evaluated by seven experts across three dimensions clarity, appropriateness and relevance and CVC values were calculated for each item. A cut-off of 0.80 was adopted as the criterion for acceptable validity (25), and inter-expert consistency was further examined using standard deviation and coefficient of variation.

**Table 2:** Content validity coefficients for questionnaire items evaluating PEST factors affecting the preparation of the Ukrainian Olympic team for games of the XXXII olympiad in Tokyo.

Factors	Item	Clarity	Appropriateness	Relevance
Political factors	1	0.893	0.893	0.893
	2	0.893	0.964	0.929
	3	0.821	0.821	0.821
	4	0.679**	0.786*	0.786*
	5	0.893	0.893	0.786*
	6	0.893	0.964	0.964
	7	0.929	0.929	0.929
Economic factors	8	0.929	0.929	0.929
	9	0.964	0.964	0.964
	10	0.929	0.929	0.929
	11	0.786*	0.893	0.893
	12	0.929	1.000	1.000
	13	0.786*	0.786*	0.786*
	14	0.929	0.929	0.929
Social-cultural factors	15	0.857	0.786*	0.786*
	16	0.893	0.893	0.893
	17	0.857	0.714*	0.857
	18	0.857	0.929	0.857
	19	0.964	0.964	0.964
	20	0.786*	0.786*	0.786*
Technological factors	21	0.893	0.964	0.893
	22	0.929	0.857	0.929
	23	0.893	0.964	0.893
	24	0.857	0.929	0.929
	25	0.893	0.929	0.929
	26	0.821	0.821	0.821
	27	0.893	0.929	0.929
	**CVC mean**	**0** **.** **87**	**0** **.** **89**	**0** **.** **89**
	SD	0.063	0.073	0.064
	CV, %	7.28	8.25	7.25

* Asterisks (*) indicate items with dimension rated below the acceptability threshold of 0.80.

** Double asterisks (**) mark items with clarity scores substantially below the threshold (CVC < 0.70), highlighting the need for substantial revision.

Bold values indicate mean CVC scores for each evaluation dimension.

The mean CVC values indicated high content validity across the instrument (clarity: 0.87; appropriateness: 0.89; relevance: 0.89), with low variability among experts (SD: 0.063—0.073; CV: 7.25%—8.25%). Seven items, however, did not meet the 0.80 threshold in at least one dimension. Item 4 showed the lowest clarity (0.679) and marginal scores on the other criteria, while Items 13 and 20 recorded sub-threshold values across all dimensions (0.786). Items 5, 11, 15 and 17 present borderline values. These findings point to issues in wording and conceptual alignment requiring further revision.

#### 3.1.1 Cognitive interviewing and item revision

To address items with sub-threshold content validity (CVC < 0.80), targeted cognitive interviews were conducted with three experts from the original validation panel. Using an online think-aloud protocol, experts reviewed the problematic items (4, 5, 11, 13, 15, 17 and 20) and verbalized their interpretation and points of confusion. Each session lasted 30—40 min. Thematic coding of the transcripts revealed recurring issues of ambiguity and weak construct alignment, which informed the rewording of these items. Table 3 presents the original formulations and their revised versions.

**Table 3:** Item revisions based on cognitive interviewing feedback.

Item	Original Wording	Revised Wording
4	Were there any international political factors (sanctions, diplomacy, IOC support) regarding Ukrainian sports?	To what extent did foreign policy factors (e.g., changes in international relations, diplomatic initiatives, sanctions, and IOC decisions) influence the preparation of Ukrainian athletes for the Games of the XXXII Olympiad in Tokyo?
5	Did Ukrainian athletes experience pressure or political discrimination during their preparation and participation in the XXXII Games of the Olympiad in Tokyo?	To what extent did political decisions related to quarantine restrictions, sports facility closures, and international event cancellations in 2020 affect the preparation of the Ukrainian Olympic team for the Games of the XXXII Olympiad in Tokyo?
11	Was there access to modern sports equipment, gear, and sports facilities during the preparation the Games of the XXXII Olympiad in Tokyo?	How adequately was the National Olympic Team of Ukraine provided with modern sports equipment, gear, and training infrastructure from a financial perspective during the preparation for the Games of the XXXII Olympiad in Tokyo?
13	Was there financial support from international organizations (IOC, European Sports Foundations, etc.) for athletes of the National Team of Ukraine in Olympic sports in preparation for the XXXII Games of the Olympiad in Tokyo?	Did Ukrainian athletes or sports federations receive financial or organizational support from international sports organizations (e.g., the IOC, Olympic Solidarity, or European sports associations) during their preparation for the Games of the XXXII Olympiad in Tokyo?
15	How did the psychological state of athletes (stress due to the pandemic) affect the results of the Ukrainian National Olympic Sports Team's performances in Tokyo?	How did social isolation, reduced public exposure, and limited interaction with families and the media in 2020–2021 affect the psychological state of Ukrainian athletes during their preparation for the Games of the XXXII Olympiad in Tokyo?
17	How important was the moral and willpower factor in the preparation and performance of Ukrainian athletes at the Games of the XXXII Olympiad in Tokyo?	To what extent did intrinsic motivation, self-discipline, and the ability to cope with stress serve as key psychological factors in the performance of Ukrainian athletes at the Games of the XXXII Olympiad in Tokyo amid the COVID-19 pandemic?
20	How strong was the support of the Ukrainian National Olympic Sports Team during its performance at the Games of the XXXII Olympiad in Tokyo from the media (social networks, journalists)?	How important was informational support from national media, social networks, and journalism in sustaining the morale of Ukrainian athletes during the Games of the XXXII Olympiad in Tokyo?

#### 3.1.2 Second round of expert validation for the reviewed items

Following revision, the updated items were re-evaluated by the original expert panel. As shown in Table 4, all reviewed items achieved CVC values between 0.821 and 1 across clarity, appropriateness and relevance. The mean post-revision CVC were 0.93—0.97, with very low variability [SD (0.05–0.56) and CV (5.17–7.02)], indicating strong expert consensus. These results confirm that the revised items reached satisfactory clarity and conceptual alignment. As only a small subset required modification, the overall questionnaire CVC was not recalculated. The instrument assessing PEST factors affecting the preparation of the Ukrainian Olympic Team for the Games of the XXXII Olympiad (Tokyo 2020) was therefore deemed to have satisfactory content validity.

**Table 4:** Content validity coefficients for the reviewed items.

Factors	Item	Clarity	Appropriateness	Relevance
Political factors	4	0.893	0.893	1.000
	5	1.000	1.000	1.000
Economic factors	11	0.929	1.000	1.000
	13	1.000	0.929	0.929
Social-cultural factors	15	0.893	0.821	0.857
	17	1.000	0.893	1.000
	20	0.964	0.964	1.000
	**CVC mean**	**0** **.** **95**	**0** **.** **93**	**0** **.** **97**
	SD	0.050	0.065	0.056
	CV, %	5.17	7.02	5.80

Bold values indicate mean CVC scores for each evaluation dimension.

### 3.2 Content validity of the questionnaire for the games of the XXXIII olympiad (Paris 2024)

Table 5 presents the content validity results for the second questionnaire. Mean CVC values were high across all dimensions (clarity: 0.89; appropriateness: 0.90; relevance: 0.91), and most items exceeded the 0.80 acceptability threshold. A small number of items showed reduced validity: Item 8 fell below the clarity threshold (0.679), while Items 12 and 28 demonstrated borderline clarity and relevance (0.786). Despite these exceptions, overall variability was low (CV = 5.38–8.37%), indicating consistent expert judgement. The questionnaire therefore demonstrates strong content validity, with only minor refinements recommended.

**Table 5:** Content validity coefficients for questionnaire items evaluating PEST factors affecting the preparation of the Ukrainian Olympic team for games of the XXXIII olympiad in Paris.

**Factors**	**Item**	**Clarity**	**Appropriateness**	**Relevance**
Political factors	1	0.857	0.964	0.964
	2	0.893	0.929	0.929
	3	0.964	0.929	0.964
	4	0.821	0.857	0.857
	5	0.893	0.893	0.893
	6	0.80	0.857	0.893
	7	1.000	1.000	1.000
Economic factors	8	0.679**	0.857	0.857
	9	0.893	0.893	0.893
	10	0.929	0.893	0.893
	11	0.857	0.964	0.964
	12	0.786*	0.821	0.786*
	13	1.000	1.000	1.000
	14	0.929	0.929	0.929
Social-cultural factors	15	0.929	0.857	0.857
	16	0.929	0.893	0.893
	17	1.000	0.893	1.000
	18	0.893	0.893	0.893
	19	0.893	0.893	0.893
	20	0.929	0.893	0.893
	21	0.964	0.964	0.964
Technological factors	22	0.821	0.857	0.821
	23	0.893	0.893	0.893
	24	0.893	0.964	0.893
	25	0.821	0.857	0.857
	26	0.893	0.929	1.000
	27	0.917	0.929	0.929
	28	0.786*	0.821	0.786*
	**CVC mean**	**0** **.** **89**	**0** **.** **90**	**0** **.** **91**
	SD	0.075	0.049	0.06
	CV, %	8.37	5.38	6.64

Asterisks (*) indicate items with dimension rated below the acceptability threshold of 0.80. Double asterisks (**) mark items with clarity scores substantially below the threshold (CVC < 0.70), highlighting the need for substantial revision.

Bold values indicate mean CVC scores for each evaluation dimension.

#### 3.2.1 Cognitive interviewing and item revision

Targeted cognitive interviews were conducted to refine items with sub-threshold validity (CVC < 0.80). Feedback indicated issues of clarity and conceptual alignment, leading to revisions of Items 8, 12 and 28. Table 6 presents the original and revised formulations.

**Table 6:** Item revisions based on cognitive interviewing feedback.

Item	Original Wording	Revised Wording
8	How did the level of state funding for sports in 2022–2024 affect the results of the National Olympic Sports Team of Ukraine at the XXXIII Games of the Olympiad in Paris?	To what extent did the level of state funding in the Olympic cycle (2021–2024) ensure the full preparation of athletes for the Games of the XXXIII Olympiad in Paris?
12	How has martial law affected the ability of Ukrainian athletes to participate in commercial tournaments?	How did martial law and restrictions on travel abroad affect the participation of Ukrainian athletes in professional (commercial) international competitions involving remuneration, contracts, or sponsorship in 2022–2024?
28	Did Ukrainian coaches have access to international conferences, seminars, and internships during the preparation for the XXXIII Games of the Olympiad under martial law?	Evaluate the impact of international educational initiatives (seminars, internships, conferences) on the work of the coaching staff during the preparation for the Games of the XXXIII Olympiad in Paris

#### 3.2.2 Second round of expert validation for the reviewed items

The revised items were re-evaluated by the same expert panel. As shown in Table 7, all items achieved substantially improved CVC values (0.821–1) across clarity, appropriateness and relevance. The mean post-revision CVC was between 0.89 and 0.96, with minimal variability (SD between 0.035 and 0.094; CV between 3.70% and 10.5%), indicating strong consensus. Because only a small subset required modification, overall questionnaire validity was not recalculated. These results confirm that the revisions effectively resolved earlier weaknesses and that the instrument demonstrates satisfactory content validity for assessing PEST-related factors influencing Olympic preparation under crisis conditions.

**Table 7:** Content validity coefficients for the reviewed items.

**Factors**	**Item**	**Clarity**	**Appropriateness**	**Relevance**
Economic factors	8	0.821	1.000	1.000
	12	0.857	0.929	0.964
Technological factors	28	1.000	0.929	0.929
	**CVC mean**	**0** **.** **89**	**0** **.** **95**	**0** **.** **96**
	SD	0.094	0.041	0.035
	CV, %	10.5	4.33	3.70

Bold values indicate mean CVC scores for each evaluation dimension.

## 4 Discussion

The developed PEST-based questionnaires offer a structured way to monitor how external crises affect elite sport. Unlike performance metrics that focus narrowly on results, these tools help reveal wider disruptions in the sport ecosystem. During the COVID-19 pandemic and the Russian invasion of Ukraine, Olympic systems faced serious external pressures—closure of facilities, displaced athletes, interrupted logistics, and reduced access to resources (7, 8, 12–14). In such situations, understanding political, economic, socio-cultural, and technological influences becomes essential. The proposed instruments support this need by capturing macro-level pressures that shape elite sport in crisis contexts.

The study also demonstrates how the PEST framework can be operationalized for empirical assessment within elite sport. While political, economic, socio-cultural, and technological influences are increasingly acknowledged in sport management and elite sport systems research (1, 3, 15), macro-environmental analysis in sport is often applied descriptively rather than through validated measurement tools (17–19). The proposed instruments translate these domains into measurable indicators, enabling the systematic assessment of macro-environmental pressures affecting Olympic preparation under crisis conditions.

Given the panel size (*n* = 7), the Content validity coefficient was used to quantify expert agreement. Unlike Lawshe's CVR, which requires large samples to reach statistical stability (24, 29, 30), the CVC is better suited for small panels and offers a continuous metric adjusted for chance agreement (23, 31). It is widely used in applied research for content validation during initial instrument development (25, 32).

Including experts from Germany, Lithuania, Poland, and Ukraine added critical perspective to the evaluation process. Their diverse backgrounds helped identify linguistic and cultural ambiguities in questionnaire items. Several items were adjusted based on this input. Through iterative refinement and cognitive interviews, conceptual clarity was improved. The revised items showed strong expert agreement, as reflected in high CVC values and very low variability—consistent with accepted benchmarks (33, 34).

The developed PEST-based instruments were designed to assess macro-environmental factors influencing Olympic athletes' preparation across two Olympic cycles (Tokyo 2020 and Paris 2024). The content-validated instruments will be applied to evaluate the impact of political, economic, socio-cultural, and technological factors on athlete preparation within the Ukrainian Olympic sport system. This enables a systematic analysis of how external crises shape elite sport over time. The proposed approach can also be adapted for use in other national sport systems operating under comparable crisis conditions. Although the specific items reflect the Ukrainian context, the underlying PEST structure may provide a useful framework for developing similar assessment tools in other countries. Such adaptation would allow researchers and sport organizations to identify context-specific external pressures while maintaining a common analytical framework.

## 5 Limitation

This study has several limitations. First, validation was restricted to expert-panel content validity. Additional psychometric evaluation commonly used in instrument development, including construct validity, reliability assessment, factor analysis, field testing, and test-retest procedures, was not conducted. Second, the instruments were developed within the context of the Ukrainian Olympic sport system during crisis conditions, which may limit direct transferability to other national settings. Future research should therefore focus on broader psychometric evaluation, pilot application, and cross-context adaptation of the instruments.

## 6 Conclusions

This study confirmed the content validity of two questionnaires developed to assess PEST influences on the preparation of the Ukrainian National Olympic Team for the XXXII and XXXIII Olympiads. Through a multi-phase validation process, combining expert review, item-level evaluation and targeted cognitive interviewing, the instruments demonstrated consistently high CVC values (0.89–0.91) across clarity, appropriateness and relevance. Most items surpassed the 0.80 threshold, and those initially below this criterion were effectively revised, yielding markedly improved validity and exceptionally low variability during re-evaluation. These results provide strong evidence that the instruments possess the semantic precision and conceptual alignment required for content-based assessment of macro-environmental factors affecting Olympic preparation under crisis conditions.

## Data Availability

The datasets generated during the current study are available from the corresponding author on reasonable request.
