# Supplementary material for: Development and validation of a PEST-based instrument for assessing macro-environmental factors in Olympic athletes' preparation under crisis conditions
Source: Front Sports Act Living. 2026 Jun 25;8:1856666. doi: 10.3389/fspor.2026.1856666 (PMC13345861; doi:10.3389/fspor.2026.1856666)
Supplement: Supplementary file 1 [file Table1.docx]

Supplementary Material

# APPENDIX A

Questionnaire Items Evaluating PEST Factors Affecting the Preparation of the Ukrainian Olympic Team for Games of the XXXII Olympiad in Tokyo.

| Items |  |  |
| --- | --- | --- |
| Political factors | | |
| 1 | 1 | To what extent did the political situation in Ukraine in 2020 (presidential elections in Ukraine, changes in the government, personnel appointments, administrative uncertainty) affect the preparation of the national team of Ukraine in summer Olympic sports for the Games of the XXXII Olympiad in Tokyo? |
| 2 | 2 | Determine the effectiveness of state policy to support athletes (programs, initiatives, strategic documents) during the preparation for the Games of the XXXII Olympiad in Tokyo? |
| 3 | 3 | How did the key strategic documents of the state policy in the field of sports (in particular, the Strategy for the Development of Physical Culture, Youth Policy, etc.) affect the preparation of the national team of Ukraine in summer Olympic sports for the Games of the XXXII Olympiad? |
| 4 | 4 | To what extent did foreign policy factors (e.g., changes in international relations, diplomatic initiatives, sanctions, and IOC decisions) influence the preparation of Ukrainian athletes for the Games of the XXXII Olympiad in Tokyo? |
| 5 | 5 | To what extent did political decisions related to quarantine restrictions, sports facility closures, and international event cancellations in 2020 affect the preparation of the Ukrainian Olympic team for the Games of the XXXII Olympiad in Tokyo? |
| 6 | 6 | How did the IOC's decision to postpone the Games of the XXXII Olympiad in Tokyo to 2021 and the related political and organizational mechanisms (changing the calendar of qualifications, adaptation of regulatory requirements, interstate agreements) affect the preparation of Ukrainian athletes for participation in these Games? |
| 7 | 7 | How did Ukraine's diplomatic actions (including cooperation with the IOC, participation in international negotiations, official statements) affect the protection of the rights of Ukrainian athletes and ensuring their participation in the Games of the XXXII Olympiad in Tokyo? |
| Economic factors | | |
| 8 | 1 | What was the impact of the level of state funding in the Olympic cycle (2016–2020) on ensuring the full preparation of athletes for the Games of the XXXII Olympiad in Tokyo? |
| 9 | 2 | What impact did individual state payments to athletes have on their preparation for the Games of the XXXII Olympiad in Tokyo? |
| 10 | 3 | What impact did the sequester (reduction) of the state budget and the associated funding restrictions during the COVID-19 pandemic have on the preparation of Ukrainian athletes for the Games of the XXXII Olympiad in Tokyo? |
| 11 | 4 | How adequately was the National Olympic Team of Ukraine provided with modern sports equipment, gear, and training infrastructure from a financial perspective during the preparation for the Games of the XXXII Olympiad in Tokyo |
| 12 | 5 | What impact did the quarantine restrictions related to the COVID-19 pandemic have on restricting the ability of Ukrainian athletes to participate in commercial tournaments in preparation for the Games of the XXXII Olympiad? |
| 13 | 6 | Did Ukrainian athletes or sports federations receive financial or organizational support from international sports organizations (e.g., the IOC, Olympic Solidarity, or European sports associations) during their preparation for the Games of the XXXII Olympiad in Tokyo? |
| 14 | 7 | To determine the degree of expenses for business trips/logistical support of the national team of Ukraine in summer Olympic sports during the preparation for the Games of the XXXII Olympiad in Tokyo? |
| Social-cultural factors | | |
| 15 | 1 | How did social isolation, reduced public exposure, and limited interaction with families and the media in 2020–2021 affect the psychological state of Ukrainian athletes during their preparation for the Games of the XXXII Olympiad in Tokyo? |
| 16 | 2 | What impact did the support from society and fans ( including live communication, online communication, public recognition) have on the moral and psychological state of athletes during the Games of the XXXII Olympiad in Tokyo? |
| 17 | 3 | To what extent did intrinsic motivation, self-discipline, and the ability to cope with stress serve as key psychological factors in the performance of Ukrainian athletes at the Games of the XXXII Olympiad in Tokyo amid the COVID-19 pandemic? |
| 18 | 4 | What impact did quarantine restrictions in 2020-2021 (cancellation of competitions, restrictions on movement, distance training) have on the preparation of Ukrainian athletes for the Games of the XXXII Olympiad in Tokyo? |
| 19 | 5 | What impact did the reduction in interpersonal interaction (team cohesion, joint meetings, restrictions on social life) have on the morale of the national team of Ukraine during the preparation for the Games of the XXXII Olympiad in Tokyo? |
| 20 | 6 | How important was informational support from national media, social networks, and journalism in sustaining the morale of Ukrainian athletes during the Games of the XXXII Olympiad in Tokyo? |
| Technological factors | | |
| 21 | 1 | To assess the level of access of the national team of Ukraine in summer Olympic sports to modern training technologies during the preparation for the Games of the XXXII Olympiad in Tokyo |
| 22 | 2 | To assess the level of access of the national team of Ukraine to the systems of physical condition monitoring, functional testing and scientific analysis of training load during preparation for the Games of the XXXII Olympiad in Tokyo? |
| 23 | 3 | To evaluate the level of technical equipment of the training bases used by the national team of Ukraine (for example, the presence of video analysis systems, sensor platforms, load monitoring devices, etc.) in preparation for the Games of the XXXII Olympiad in Tokyo. |
| 24 | 4 | To evaluate how actively and systematically modern research equipment (motion analyzer, test platforms, etc.) was used in the preparation of athletes for the Games of the XXXII Olympiad in Tokyo? |
| 25 | 5 | To assess the extent to which uninterrupted access of Ukrainian athletes to sports medicine services, rehabilitation procedures and rehabilitation support was ensured during the preparation for the Games of the XXXII Olympiad in Tokyo, in the context of quarantine restrictions? |
| 26 | 6 | To assess how actively and effectively digital technologies (virtual training, online communication, digital monitoring) were used in the preparation of Ukrainian athletes for the Games of the XXXII Olympiad in Tokyo? |
| 27 | 7 | To assess the impact of international educational initiatives (seminars, internships, conferences) on the work of the coaching staff in preparation for the Games of the XXXII Olympiad in Tokyo. |

# APPENDIX B

Questionnaire Items Evaluating PEST Factors Affecting the Preparation of the Ukrainian Olympic Team for Games of the XXXIII Olympiad in Paris.

| Items |  |  |
| --- | --- | --- |
| Political factors | | |
| 1 | 1 | How did hostilities and martial law in Ukraine affect the training of athletes of the national team of Ukraine in summer Olympic sports in 2022-2024? |
| 2 | 2 | What was the impact of the state programs to support the national team of Ukraine in summer Olympic sports in 2022-2024 to ensure the proper level of preparation for the Games of the XXXIII Olympiad in Paris? |
| 3 | 3 | What impact did the forced relocation of athletes and training bases (in Ukraine or abroad) as a result of the war have on the quality of preparation of the national team of Ukraine for the Games of the XXXIII Olympiad in Paris? |
| 4 | 4 | What was the impact of international politics (in particular, the decision of the IOC, the boycott of Russian and Belarusian athletes, international solidarity) on the preparation and performance of the national team of Ukraine at the Games of the XXXIII Olympiad in Paris? |
| 5 | 5 | What was the impact of the political atmosphere in relation to Ukrainian athletes (cases of political pressure, discrimination, etc.) during their participation in the Games of the XXXIII Olympiad in Paris? |
| 6 | 6 | What was the impact of the level of diplomatic support and protection of the interests of the national team in summer Olympic sports during the preparation in 2022-2024? |
| 7 | 7 | How did international cooperation (through the NOC, IOC, federations) affect the organization of training camps for Ukrainian athletes abroad in 2022-2024? |
| Economic factors | | |
| 8 | 1 | To what extent did the level of state funding in the Olympic cycle (2021–2024) ensure the full preparation of athletes for the Games of the XXXIII Olympiad in Paris? |
| 9 | 2 | What was the impact of individual financial support (scholarships, grants, awards) of the athletes of the national team of Ukraine in summer Olympic sports during 2021-2024? |
| 10 | 3 | What impact did the war in Ukraine have on the possibilities of attracting financial support from private businesses (companies, commercial sponsors) for the national team of Ukraine in summer Olympic sports in 2022-2024? |
| 11 | 4 | To assess how the national Olympic team of Ukraine was provided with modern sports equipment, equipment and training infrastructure from a financial point of view in the process of preparation for the Games of the XXXIII Olympiad in Paris? |
| 12 | 5 | How did martial law and restrictions on travel abroad affect the participation of Ukrainian athletes in professional (commercial) international competitions involving remuneration, contracts, or sponsorship in 2022–2024? |
| 13 | 6 | How did financial or organizational support from international sports organizations (IOC, Olympic Solidarity, European federations, etc.) affect the effectiveness of the preparation of the national team of Ukraine in summer Olympic sports in 2022-2024? |
| 14 | 7 | How did the wartime funding affect the costs of business trips, accommodation and logistics during the preparation of the national team of Ukraine in summer Olympic sports for the Games of the XXXIII Olympiad in Paris? |
| Social-cultural factors | | |
| 15 | 1 | How did the psychological state of Ukrainian athletes (stress, anxiety, uncertainty due to military events) affect their performance during the Games of the XXXIII Olympiad in Paris? |
| 16 | 2 | What was the impact of the support of society and fans during the performance of Ukrainian athletes at the Games of the XXXIII Olympiad in Paris? |
| 17 | 3 | How did the moral and volitional factor associated with the war ("fight for the country", national responsibility) affect the motivation of athletes during the preparation and participation in the Games of the XXXIII Olympiad in Paris? |
| 18 | 4 | What was the impact of the forced movement of athletes (within Ukraine or abroad) in connection with hostilities on the quality of their social adaptation, communication and preparation for the Games of the XXXIII Olympiad in Paris? |
| 19 | 5 | How the limited communication of the athletes of the national team of Ukraine in summer Olympic sports with their families during the preparation for the Games of the XXIII Olympiad in Paris affected their psychological state? |
| 20 | 6 | How did the media support (social networks, journalists) of the national team of Ukraine in summer Olympic sports affect during the preparation and participation in the Games of the XXXIII Olympiad in Paris? |
| 21 | 7 | What was the impact of cases of discrimination against Ukrainian athletes due to the war during the preparation and participation in the Games of the XXXIII Olympiad in Paris? |
| Technological factors | | |
| 22 | 1 | To assess the level of access of the national team of Ukraine in summer Olympic sports to modern training technologies during the war in preparation for the Games of the XXXIII Olympiad in Paris? |
| 23 | 2 | To assess how the war has affected the level of access of the national Olympic team of Ukraine to systems for monitoring physical condition, functional testing and scientific analysis of training load in preparation for the Games of the XXXIII Olympiad in Paris? |
| 24 | 3 | To assess the level of technical equipment of the training bases used by the national team of Ukraine (for example, the availability of video analysis systems, sensor platforms, load monitoring devices, etc.) during the preparation for the Games of the XXXIII Olympiad in Paris under martial law? |
| 25 | 4 | To assess how actively and systematically modern research equipment (motion analyzer, test platforms, etc.) was used in the preparation of athletes for the Games of the XXXIII Olympiad in Paris under martial law? |
| 26 | 5 | To assess the degree of access of Ukrainian athletes to sports medicine services, recovery procedures and rehabilitation support during the preparation for the Games of the XXIII Olympiad under martial law? |
| 27 | 6 | To assess how actively and effectively digital technologies (virtual training, online communication, digital monitoring) were used in the preparation of Ukrainian athletes for the Games of the XXXIII Olympiad in Paris under martial law? |
| 28 | 7 | To evaluate the impact of international educational initiatives (seminars, internships, conferences) on the work of the coaching staff during the preparation for the Games of the XXXIII Olympiad in Paris |
